# Increasing mobility limitations among older adults in Finland: time trends and projections during 2013–2040

**DOI:** 10.1186/s12889-026-28941-6

**Published:** 2026-08-10

**Authors:** Marja Eliisa Holm, Jaakko Reinikainen, Päivi Sainio, Katri Sääksjärvi, Annamari Lundqvist, Jouni Lahti, Seppo Koskinen, Suvi Parikka

**Affiliations:** Department of Public Health, Finnish Institute for Health and Welfare, P.O. Box 30, Helsinki, FI-00271 Finland

**Keywords:** Education level, Mobility limitations, Past and future trends, Older adults, Population-based study

## Abstract

**Background:**

In an ageing population, physical functioning is a major public health issue. Limited information exists on how mobility limitations will change as the population ages. We assessed past and future trends in mobility limitations among the population aged 55 and older in Finland from 2013 to 2040 by age group and educational level.

**Methods:**

We analyzed seven representative cross-sectional surveys in 2013, 2014, 2015, 2018, 2020, 2022, and 2024 (*n* = 132,427, aged 55+). Self-reported mobility limitations were defined as major difficulty or an inability to walk approximately half a kilometer. Multiple imputation was used to project future mobility limitations based on the most recent population projection by Statistics Finland.

**Results:**

The observed and projected increasing time trend in the prevalence of mobility limitations from 2013 to 2040 was the most pronounced among the population aged 75 and older, while it remained stable among younger groups (55–64, 65–74). Following a slow decline from 2013 to 2018, the prevalence of limitations increased from 2018 (23%) to 2020 (29%) among persons aged 75+, remained high until 2024 (27%), and is projected to reach 35% by 2040. Due to the increasing prevalence of mobility limitations and the growing number of older people, the number of persons with limitations increased more than 50% between 2018 (117,000) and 2024 (177,000), with the sharpest increase observed between 2018 and 2020. This number is projected to nearly double by 2040, reaching 306,000 among persons aged 75 years or older. Across all educational groups among persons aged 55+, the prevalence of limitations remained stable from 2013 to 2018. In the low- and medium-educated groups, the prevalence increased from 2018 to 2020 and then remained stable until 2024, while in the high-education group it increased steadily from 2018 to 2024. Projections indicate a slight rise in all educational groups by 2040.

**Conclusions:**

Mobility limitations are projected to increase, especially in the population aged 75 or older, by 2040. Effective preventive actions, such as improved support and opportunities for physical activity and balanced healthy nutrition, are needed to slow down the rapid increase in mobility limitations. Moreover, improving accessibility enables people with mobility limitations to cope independently in society.

**Supplementary Information:**

The online version contains supplementary material available at https://doi.org/10.1186/s12889-026-28941-6.

## Introduction

Mobility limitations can seriously affect health and well-being. Adults with mobility limitations are particularly prone to falls, and they have increased risk for future disability in the activities of daily living, increased healthcare utilization and expenditure, low self-efficacy and quality of life, and a high risk of pain and mortality [1–6]. Information on past trends and future development is essential for planning and providing adequate services for the ageing population.

There is limited research on how the prevalence of mobility limitations has changed since the beginning of the twenty-first century. Härkänen et al. showed that the prevalence of the inability to walk about half a kilometer or major difficulties in walking about half a kilometer decreased from 14.4% to 10% between 2000 and 2011 in the Finnish population aged 52 years and older [7]. Other studies also suggest a decline in self-reported mobility limitations among persons aged 65 years or older between 2008 and 2017 in the US, as well as between 2001 and 2013 in Taiwan, for both women and men [8, 9]. However, there are also opposite findings. Palacios-Ceña et al. found that self-reported mobility limitations increased slightly between 2000 and 2007 among both women and men aged 65 and older in Spain [10]. Although trends in mobility limitations appear to be similar for both sexes, a systematic review shows that such limitations are more common among women aged 60 or over than among men [11]. Nevertheless, information on time trends in mobility limitations during the most recent decade is not available.

Additionally, although previous research shows that mobility limitations increase with age [7, 12, 13], there is no certainty whether the change over time is different across age groups. Fuller-Thomson et al. found decreases in mobility limitations across all age groups (65–74, 75–84, and 85+) between 2008 and 2017 [8], while Santoni et al. reported declines in impaired walking speed between 2001 and 2016 among those aged 66–84, but not among those 85 or older [14]. Similarly, Öhman et al. observed increases in self-reported good mobility among 75-, 80-, and 85-year-olds between 1989 and 2019, but not among those aged 90 or older [15]. These results suggest that the oldest adults were less likely to show improvement in mobility.

Previous studies have assessed future time trends in limitations in the activities of daily living, the instrumental activities of daily living, and cognitive functions among older adults [16–18], but we lack studies on future time trends in mobility limitations. A Finnish study indicated a significant increase in the prevalence of and number of people with mobility limitations during the period 2011–2044 among the population aged 52 years and older [7]. Correspondingly, Smith et al. reported that the proportion of the population with self-reported mobility limitations will increase from 2000 to 2050 in the US [19]. However, these projections were based on the observed prevalences at only one time point (the year 2000; [19]) or only two time points (years 2000 and 2011; [7]), and we need more recent data when projecting the future. Population aging is a global trend, and the proportion of older adults is expected to continue rising in the future both in Finland [20] and elsewhere [21, 22].

Previous studies have found that adults with a low level of education are more likely to develop mobility limitations than those with higher education [23, 24]. A lower level of education is associated with lower occupational class and thereby poorer working conditions, which may contribute to mobility limitations across the life course. Additionally, poor health behavior and obesity—more common among those with a lower level of education—partly explain differences in physical functioning [25]. Fuller-Thomson et al. found that higher education was associated with a greater decrease in mobility limitations between 2008 and 2018 among persons aged 65 or older [8]. Additionally, a study conducted in South Korea showed that a lower level of education was associated with mobility decline during the COVID-19 pandemic [26], suggesting that it is important to investigate changes in mobility limitations by education. However, there is limited information on whether recent changes in mobility limitations over time differ by level of education.

Based on the above, we examined time trends in the prevalence of mobility limitations from 2013 to 2024 and made projections until 2040 among adults aged 55 and older. Furthermore, we examined the results according to sex, age group, and educational level.

## Methods

### Data and design

Our analysis used population-based data of adults aged 55 or older (*n* = 132,427) from multiple surveys conducted between 2013 and 2024. Table 1 presents the descriptive statistics of these surveys. The surveys included the Regional Health and Wellbeing Study (2013 (*n* = 29,310), 2014 (*n* = 11,851), 2015 (*n* = 12,269); [27]), the National FinSote Survey (2018 (*n* = 19,551) and 2020 (*n* = 20,305); [28]), and the Healthy Finland Survey (2022 (*n* = 19,730) and 2024 (*n* = 19,411); [29]). The survey design in all seven surveys was based on stratified random sampling using a sample frame from the Finnish Population Register, ensuring comparability across the years. In all surveys, individuals aged 75 and older were oversampled, with no exclusion criteria. Yet, the coverage of older adults living in institutional settings (in communal housing or long-term care) remained limited (approximately 3–4% of respondents aged 75 years and older). Thus, the majority of survey respondents, even in the 75 + age group, are older adults living in private dwellings.

**Table 1:** The study participants and descriptive statistics by study year among adults aged 55+

	2013	2014	2015	2018	2020	2022	2024
Total, *n*	29,310	11,851	12,269	19,551	20,305	19,730	19,411
Response rate, %	64.4%	64.5%	66.9%	56.1%	57.7%	57.3%	57.1%
Sex, % [95% CI] (*n*)							
Men	46.2 [45.5, 46.8] (12,657)	44.7 [43.7, 45.8] (5,085)	45.7 [44.7, 46.8] (5,355)	46.1 [48.2, 47.0] (8,620)	45.4 [45.2, 46.3] (8,980)	45.9 [44.6, 46.8] (8,799)	46.5 [45.6, 47.4] (8,551)
Women	53.8 [53.2, 54.5] (16,653)	55.3 [54.2, 56.3] (6,766)	54.3 [53.2, 55.3] (6,914)	53.9 [53.0, 54.8] (10,931)	54.6 [53.7, 55.4] (11,325)	54.1 [53.2, 55.0] (10,930)	53.5 [52.6, 54.4] (10,860)
Age, % [95% CI] (*n*)							
55–64	42.7 [42.0, 18.8] (9,704)	41.3 [40.3, 42.4] (3,748)	40.5 [39.5, 41.6] (3,862)	37.8 [36.9, 38.7] (4,507)	36.2 [35.4, 37.1] (4,616)	35.7 [34.9, 36.6] (4,551)	34.4 [33.5, 35.2] (3,941)
65–74	32.0 [31.4, 32.6] (8,503)	33.2 [32.2, 34.2] (3,502)	33.9 [32.9, 34.9] (3,692)	35.6 [34.7, 36.5] (5,263)	35.5 [34.6, 36.3] (5,770)	33.7 [32.8, 34.5] (5,492)	33.5 [32.7, 34.4] (5,078)
75+	25.3 [24.7, 25.9] (11,103)	25.4 [24.5, 26.4] (4,601)	25.6 [24.7, 26.5] (4,715)	26.6 [25.8, 27.4] (9,781)	28.3 [27.5, 29.1] (9,919)	30.6 [29.8, 31.4] (9,686)	32.1 [31.2, 32.9] (10,392)
Education, % [95% CI] (*n*)							
Low	46.7 [46.0, 47.4] (11,983)	44.1 [43.0, 45.3] (4,620)	42.7 [41.6, 43.8] (4,610)	39.7 [38.7, 40.6] (7,067)	41.7 [40.8, 42.6] (7,482)	42.8 [41.9, 43.7] (7,548)	43.3 [42.4, 44.2] (7,525)
Medium	28.4 [27.8, 29.0] (7,392)	29.1 [28.1, 30.2] (3,022)	30.2 [29.2, 31.2] (3,244)	29.5 [28.6, 30.3] (5,079)	31.8 [31.0, 32.6] (6,340)	29.6 [28.8, 30.5] (5,714)	29.3 [28.5, 30.2] (5,587)
High	24.9 [24.3, 25.5] (7,513)	26.7 [25.7, 27.7] (3,213)	27.1 [26.2, 28.1] (3,392)	30.9 [30.0, 31.8] (5,305)	26.5 [25.7, 27.3] (5,231)	27.6 [26.8, 28.4] (5,470)	27.4 [26.6, 28.2] (5,433)

The sample design was considered in the calculation of percentages

*CI* confidence interval

The surveys were carried out by the Finnish Institute for Health and Welfare (THL), and the survey protocols were approved by the THL Ethics Committee. Respondents had the option of completing the questionnaires online or on paper, and those who did not reply were subsequently contacted via phone calls and sent reminder letters or SMS messages. The response rates were between 57% and 67% across the different surveys (see Table 1).

### Measures

All survey questionnaires used in this study are publicly available and have been published by the Finnish Institute for Health and Welfare [30]. Mobility limitations were assessed with the question “Can you usually walk about 500 m without stopping to rest?” with the response options “Yes, I can with no difficulty,” “Yes, I can with some difficulty,” “Yes, I can with great difficulty,” and “I cannot” [31, 32]. The last two responses were combined to indicate mobility limitations. This walking indicator was chosen as a measure of mobility limitations because declining walking ability is often an early sign of mobility restrictions and a strong predictor of future health, functional capacity, and healthcare utilization [33], making walking difficulties a key contributor to mobility limitations.

We used the following sociodemographic covariates: register-based age (continuous) and sex (binary: female and male), as well as the level of education (categorical: low, medium, and high levels). The level of education was based on the self-reported number of years of full-time schooling or studying, including primary and secondary school (the question was: “How many years did you attend school or study full time in total?”). The relative level of education was determined by first dividing the respondents by sex into 10-year age groups and then dividing them into tertiles according to years of education (a low level of education [the lowest tertile], a medium level of education [the middle tertile], a high level of education [the highest tertile]).

### Data analysis

Survey weights were used to calculate prevalences, accounting for sampling design and non-participation. The weights were calculated using inverse probability weighting, based on register data on age, sex, marital status, education, region, and native language for the entire sample [27].

We examined time trends in mobility limitations from 2013 to 2024 and made projections until 2040 among adults aged 55 or older. R program and logistic regression models were used in the analyses. In these models, mobility limitations were the outcome variable and sociodemographic factors and survey years were the predictors. Because age was significantly associated with the outcome, it was also controlled for in age-group–specific analyses. Due to significant interactions between the age groups (55–64, 65–74, 75+) and the year (*p* < .001) and the education levels (low, medium, high levels) and the year (*p* < .001), the time trends and projections are shown by age group and education. The results are presented separately by sex due to sex differences in mobility limitations at certain time points.

The future prevalences of mobility limitations were projected using a multiple imputation approach previously applied to project the population-level prevalence of other health indicators [34–37]. In this method, the past changes in the population-based survey data are used to predict future changes by assuming that the observed time trends will continue. The model not only projects prevalence based on recent observed time trends, but also accounts for expected future changes in population age structure, where one of the factors is changes in life expectancy. The changes in population age structure are based on the population projection by Statistics Finland, in which changes in population age structure are driven by assumptions regarding mortality (life expectancy development), fertility, and net migration [20]. Next, we describe the method step by step.

#### Projection procedures

In the first step, future unobserved samples for 2027, 2030, 2035, and 2040 were created, each with an average size of 18,835 adults aged 55 and older based on the observed samples. The unobserved samples’ age and sex distributions were matched to Finland’s national population projections for those years to account for population change [20]. In the second step, the new data were combined with the observed data in the same frame, with future sample rows having fixed survey year, age, education, and sex, and missing mobility limitation values before imputation. The education variable was created for future data by dividing the data into thirds (which were assigned the values *low*,* medium*,* high*) such that each of the thirds had the same age and sex distribution. In the third step, mobility limitations for 2027, 2030, 2035, and 2040 were projected by imputing missing future values multiple times using past population data from 2013 to 2024 [38]. The missing values (item non-responses) in the observed samples were also imputed. Age, sex, and survey year had no missing data. The method was implemented using the mice package in R for multiple imputation (100 imputations and 10 iterations; [37]) with binary and polytomous logistic regressions. In the final step, imputed values were pooled with Rubin’s rules to estimate mobility limitation prevalences and standard errors [39]. Wilson intervals were used as prediction intervals for the prevalences [40].

In each projection models, we tested whether the association between survey years and the outcome variable—mobility limitations—was best modeled as a linear or a non-linear cubic spline model [41]. Imputation models were chosen based on the Bayesian Information Criterion (BIC), where lower BIC values indicate a better fit [42]. In each case, linear models were selected.

## Results

### Mobility limitations among adults aged 55+

**Fig. 1:**
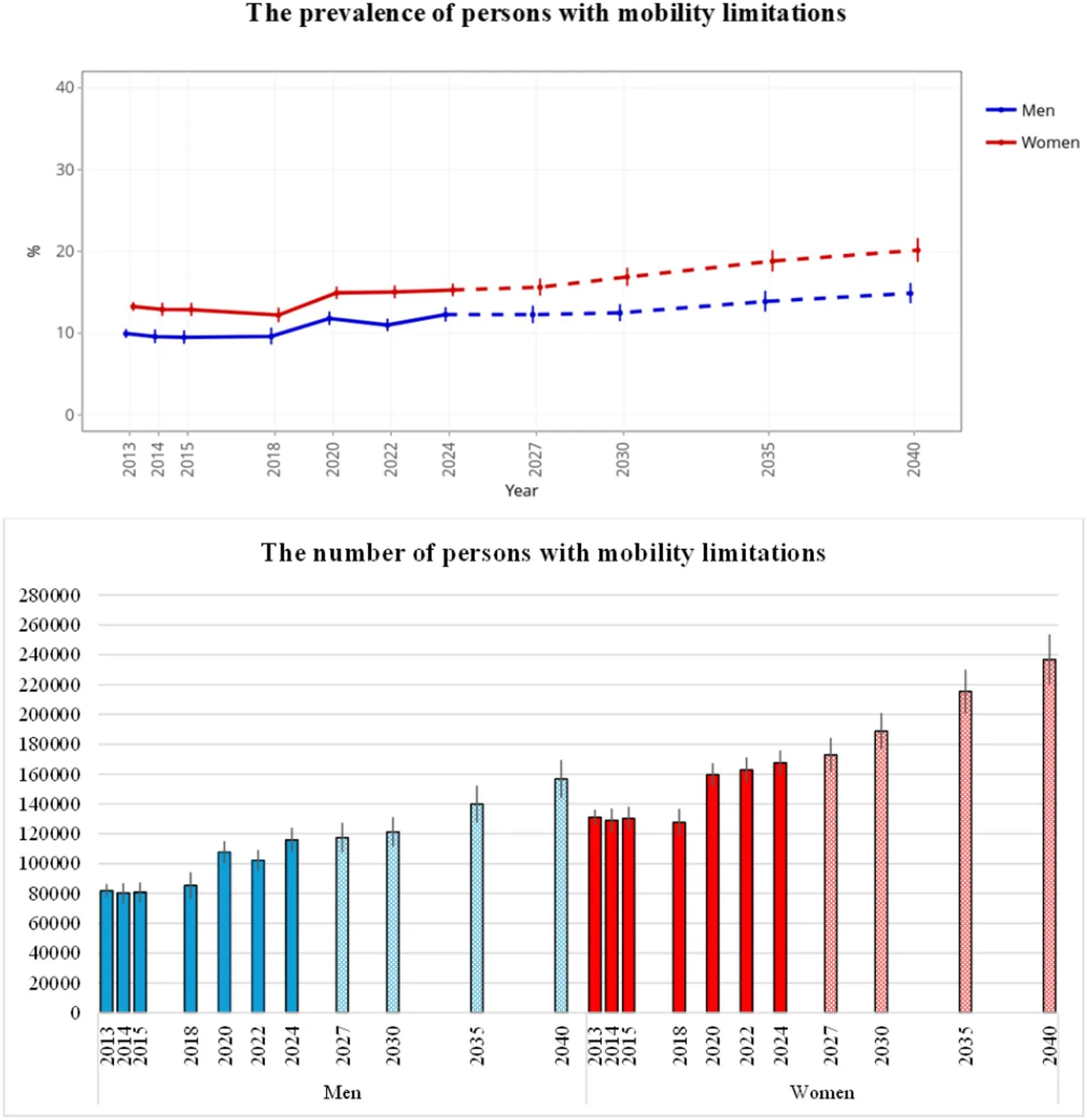
Time trends from 2013 to 2024 and projections until 2040 for the prevalence (the upper panel) and the number of persons (the lower panel) with mobility limitations among men and women aged 55 and older

The prevalence of mobility limitations among adults aged 55 and older remained stable between 2013 and 2018 for both men and women (see Fig. 1; Additional file 1: Appendix A). Between 2018 and 2020, the prevalence increased significantly in both women (2018: 12%; 2020: 15%) and men (2018: 10%; 2020: 12%) and remained high until 2024 (women: 15%; men: 12%). An increasing time trend is projected to continue, and by 2040, the prevalence of mobility limitations is estimated to be around 20% for women and 15% for men. Correspondingly, the number of people with mobility limitations increased more than 30% from 2018 to 2024 (from 85,397 to 115,885 men and from 127,777 to 167,650 women); the largest increase was observed between 2018 and 2020 (see Fig. 1; Additional file 1: Appendix B).

Due to increases in the number of older people (see Additional file 1: Appendix B) and in the prevalence of mobility limitations, the number of persons with mobility limitations is projected to increase by about 40% by 2040, being projected to reach approximately 156,761 men and 236,911 women (see Fig. 1). While we observed similar time trends and projections by sex, women had more mobility limitations than men in each year (*p* < .001), and sex differences were projected to persist up to 2040 (see Fig. 1). 

## Mobility limitations by sex and age

**Fig. 2:**
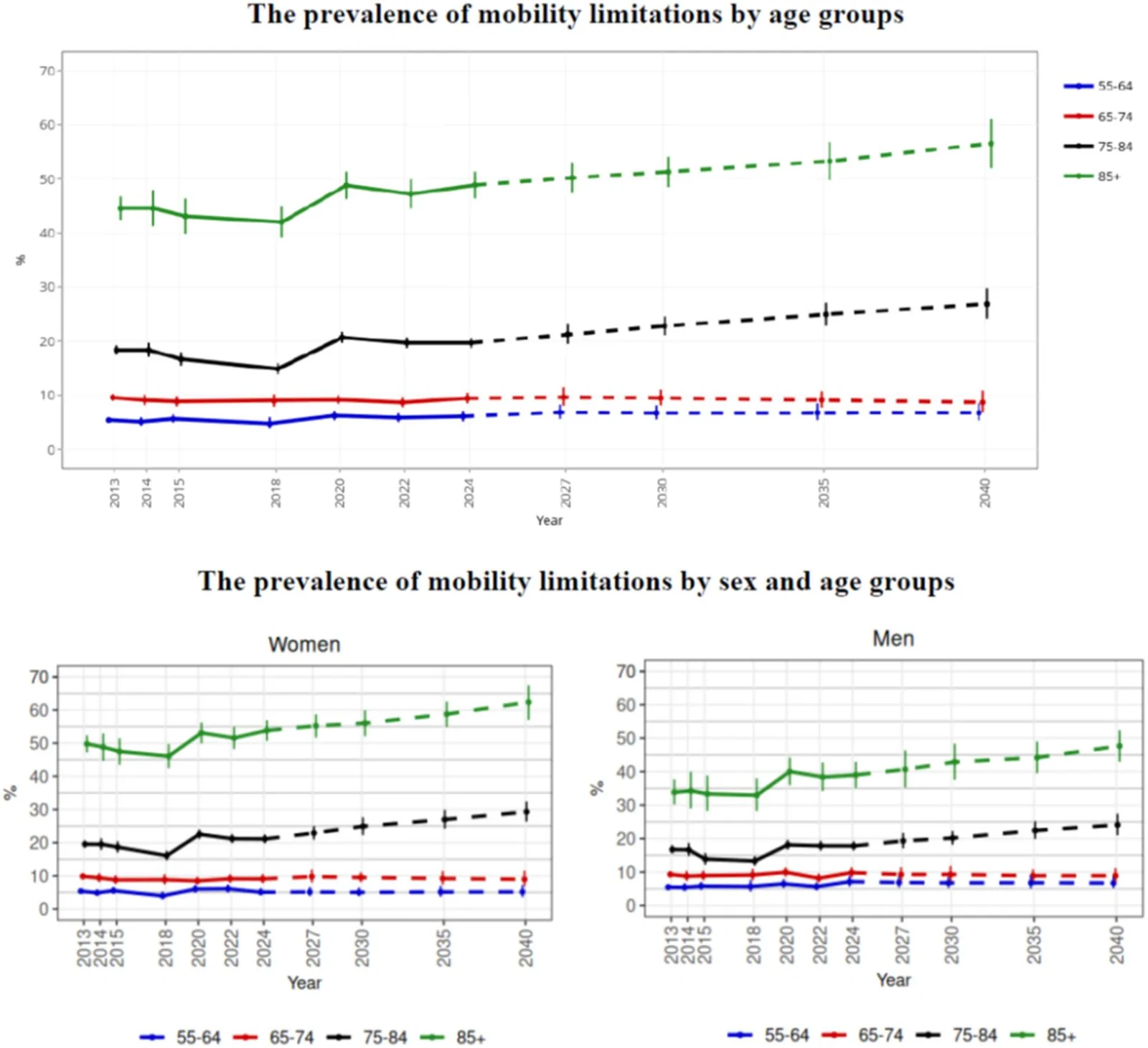
Time trends from 2013 to 2024 and projections until 2040 for the prevalence of mobility limitations by age group among adults aged 55+

Following a decreasing trend from 2013 to 2018, the prevalence of mobility limitations increased between 2018 (23%) and 2020 (29%) among people aged 75 + and remained high from 2020 to 2024 (2024: 27%). The prevalence is projected to increase in the future, being projected to reach 35% by 2040 (see Additional file 1: Appendix A). When persons aged 75 or older were divided into more detailed age groups (75–84 and 85+), the decreasing time trend between 2013 and 2018 was particularly observed among those aged 75–84 (see Fig. 2; Additional file 1: Appendix A). The observed increases from 2018 to 2024 and the projected increases by 2040 were found among both men and women aged 75–84 and 85+. In contrast, the observed and projected prevalences remained stable in the younger age groups (55–64; 65–74).

Women aged 75–84 and 85 + had significantly more mobility limitations than men of the same age (*p* < .05), whereas sex differences were not significant among the younger age groups (55–64; 65–74) at any time point. These sex differences are projected to persist during the whole period covered by the projection. The burden of mobility limitations was highest among women aged 85 and older: in 2024, over one-half of them reported mobility limitations, and this prevalence is projected to increase to 60% by 2040. 

**Fig. 3:**
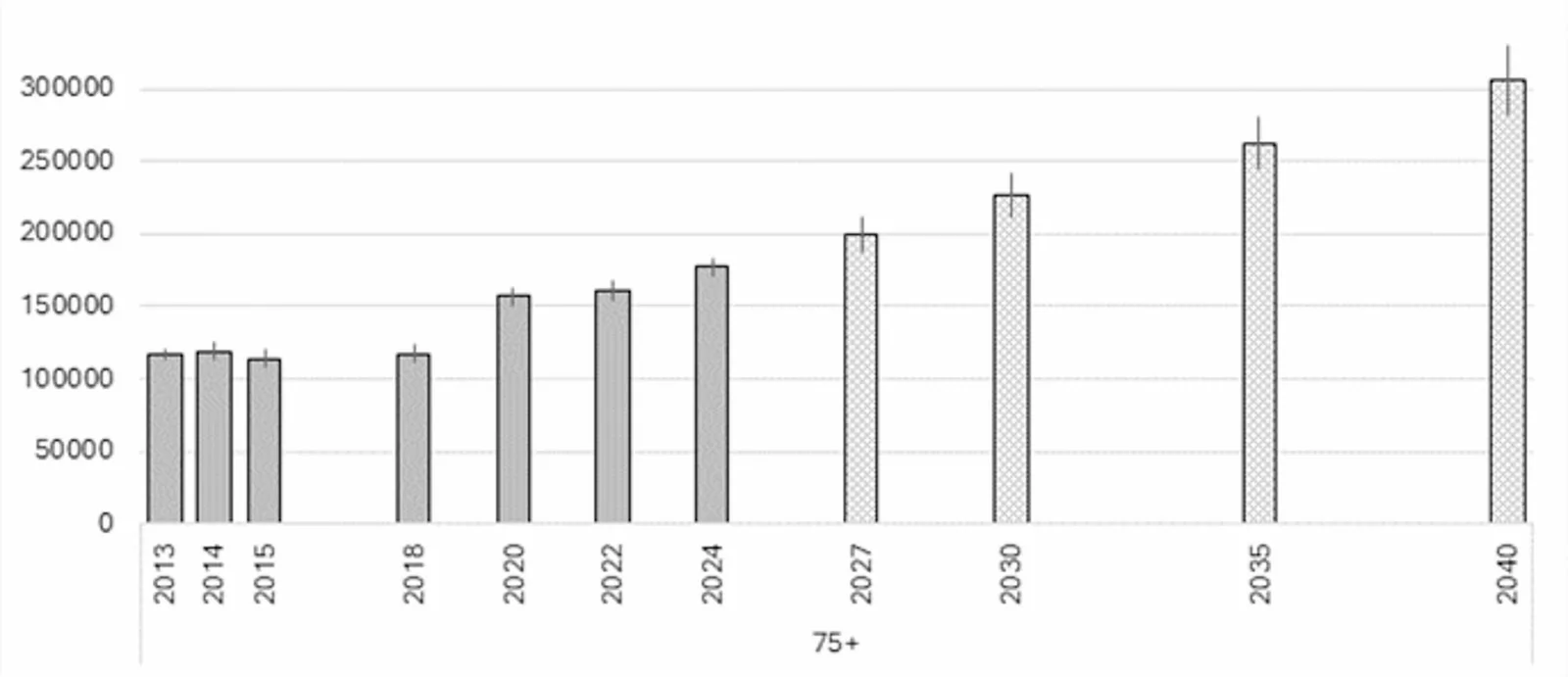
Time trends from 2013 to 2024 and projections until 2040 for the number of people with mobility limitations among persons aged 75+

Due to the increasing prevalence of mobility limitations and the increasing number of older people in the population (see Fig. 3; Additional file 1: Appendix B), the number of people aged 75 + with mobility limitations increased more than 50% from 2018 to 2024, reaching 177,058 in 2024; the largest increase occurred between 2018 and 2020, after which the rise was linear. The number of individuals with mobility limitations is projected to nearly double from 2024 to 2040, being projected to increase to 306,449 among persons aged 75 years and older in 2040. 

## Mobility limitations by level of education

**Fig. 4:**
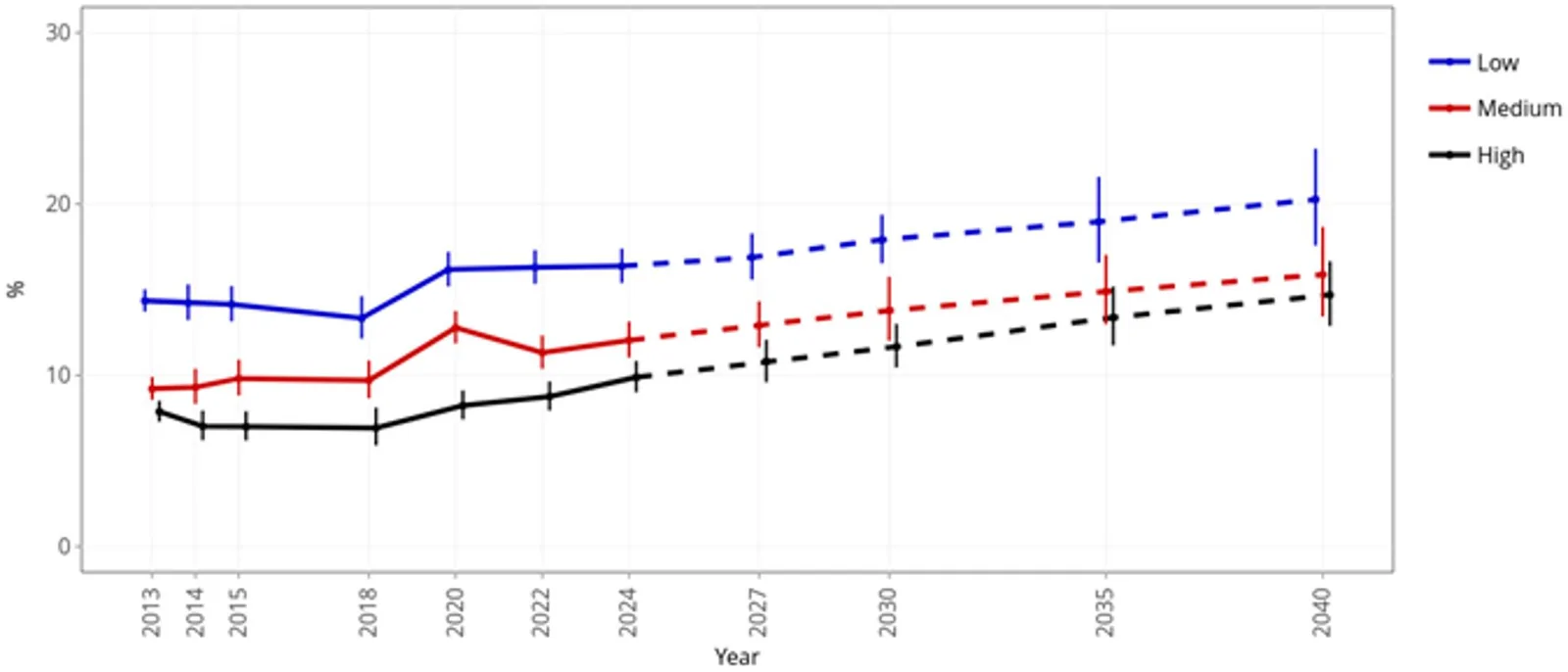
Time trends from 2013 to 2024 and projections until 2040 for mobility limitations among adults aged 55 and older by educational level

The prevalence of mobility limitations was clearly highest among people with a low level of education and lowest among people with a high level of education (see Fig. 4; Additional file 1: Appendix C). Parallel time trends and projections were observed among people with a low and medium level of education, both for men and women. No changes were observed between 2013 and 2018, but there was a clear increase from 2018 to 2020 (for those with a low level of education: from 13% to 16%; for those with a medium level of education: from 10% to 13%). Thereafter the prevalence remained at a high level (for those with a low level of education: 16%; for those with a medium level of education: 12%) until 2024. Future projections indicate a slight increase in the prevalence of mobility limitations by 2040, reaching 20% for people with a low level of education and 16% for those with a medium level of education.

Among people with a high level of education, the prevalence of mobility limitations remained stable from 2013 to 2018 but increased steadily from 2018 (7%) to 2024 (10%). The projection indicates an increasing time trend from 2024 to 2040, being projected to reach 15% by 2040. The increasing time trend between 2018 and 2024 among people with a high level of education was especially noticeable among women (2018: 8%; 2024: 12%), whereas for men, the upward trend was not statistically significant (2018: 6%; 2024: 8%; see Additional file 1: Appendix C). For both sexes, the prevalence of mobility limitations is expected to increase in the future up to 2040, with a greater increase among women. 

## Discussion

The present study showed that the prevalence of mobility limitations has increased since 2018 among the population aged 75 + but not in those aged 55–74 years. By 2040, 35% of people aged 75 years or older are projected to have a mobility limitation if limitations continue developing similarly as they have during the period from 2013 to 2024. Moreover, the projections up to 2040 indicate that the number of persons with mobility limitations is expected to nearly double by 2040, reaching 306,000 among 75-year-olds and older people, mostly due to population ageing. We also observed somewhat different changes across education groups. In the people with a low or medium level of education, the most notable increase in mobility limitations occurred between 2018 and 2020, after which the levels remained stable from 2020 to 2024, while among people with a high level of education, limitations increased steadily between 2018 and 2024. The projection showed a slight increase in the prevalence of mobility limitations in all educational groups.

While previous research has found a decrease in the prevalence of mobility limitations between 2000 and 2018 among adults 65 or older [7, 8, 9], we found a slight decrease in mobility limitations from 2013 to 2018 among persons aged 75–84 but not among those aged 55–64, 65–74, or 85+. This is partly consistent with prior research showing that improvement in mobility since 2000 has not extended to the oldest populations [14, 15]. Moreover, our results also contribute to previous research indicating that the oldest people (aged 85 years or older) are the most vulnerable to having mobility limitations [7, 12].

The sharp increase in mobility limitations among people aged 75 and over between 2018 and 2020 may be due to the restrictions caused by the COVID-19 pandemic. These restrictions reduced participation in physical activities and access to healthcare and rehabilitation services [43–45], while potentially increasing fast-food consumption and snacking among older adults [46, 47]. Such behavioral and service-related changes are likely to have especially affected older people, leading to declines in physical fitness and unmet healthcare needs that may have persisted beyond the pandemic [48–50], which may explain why the prevalence of mobility limitations has not decreased after the pandemic. Additionally, the population is aging as people live longer, increasing the proportion of older adults [21, 22], which may contribute to mobility limitations remaining high in 2024 and continuing to increase in the future. On the other hand, we observed a relatively stable trend from 2013 to 2024 among those aged 55–74, indicating no improvement in mobility limitations. This stability may reflect two concurrent factors. First, the age distribution within these groups remained largely unchanged over time, limiting demographic-driven shifts in mobility. Second, population-level trends—such as the persistently high or increasing prevalence of obesity and sedentary behavior—may worsen mobility [51, 52]. For example, recent evidence from Finland shows that among adults aged 55–64, severe obesity (BMI ≥ 35 kg/m²) increased markedly between 2000 and 2023 in both sexes, while obesity (BMI ≥ 30 kg/m²) showed no significant change [51]. Moreover, improvements in rehabilitation, medical care, and access to assistive technologies may have helped maintain physical functioning [53], despite an increasing burden of risk factors. Taken together, these opposing trends may contribute to the observed stability in mobility limitation prevalence.

In line with previous literature [8–10, 11], we observed a similar trend in mobility limitations in women and men between 2013 and 2024, although limitations were more common among women aged 75 and older than among men of the same age. However, we did not find a sex difference in the younger age groups. These age-specific sex differences in mobility limitations may reflect, for example, faster age-related muscle loss, higher fat mass, and a lower likelihood of engaging in regular sports or exercise among women than men in older age [54, 55]. Our study indicated that women aged 85 and older very often have mobility limitations: in 2024, over half of them had limitations, and this proportion is projected to exceed 60% by 2040.

We found that mobility limitations increased steadily from 2018 to 2024 among highly educated persons aged 55 and older, particularly among women, while among those with a low or medium level of education, the prevalence increased sharply between 2018 and 2020 and then remained quite stable through 2024. The prevalence is projected to increase across all education groups, suggesting that higher education does not appear to attenuate the increasing trend. People with a high level of education may be more likely to have desk jobs or work that involves prolonged sitting, which decreases physical activity and can weaken muscle strength and balance [56], potentially contributing to mobility limitations later in life. Our study suggests that highly educated women may be more susceptible to these negative factors that increase mobility limitations. On the other hand, adults with a low level of education may face barriers to accessing healthcare and rehabilitation services and are more likely to engage in unhealthy habits (such as physical inactivity and consuming nutritionally poor meals) and obesity [57–60], which may increase the risk of mobility limitations. These factors may help explain the observed sharp increase in mobility limitations between 2018 and 2020 among those with low or medium level of education. Although this rise appears to have stabilized after restrictions were lifted, prevalence among adults with a low or medium level of education has remained high and is projected to increase slightly in the future. Consistent with previous research, our findings highlight persistent educational inequalities in mobility limitations [23, 24], while highlighting the need for preventive intervention across all educational groups. Notably, the suggested mechanisms underlying changes in mobility limitations by educational level are interpretive, and further research is needed to clarify the factors driving these trends.

### Limitations and strengths

A strength of our study is that the data were based on population-based surveys of random samples of the general population. An additional benefit of our research is its large sample size, which represents the older adult population in Finland. Despite the relatively high response rate and the adjustment for nonresponses through survey weights based on comprehensive register data for all sample members, our data may still be impacted on by possible selection bias due to nonresponses. Individuals with poor health are often unable to answer self-administered questionnaires. To better include these groups, which are underrepresented in national health surveys, interviews should be conducted in the respondent’s home or other dwelling, thereby reducing physical, cognitive, and mobility-related barriers. At the same time, information is also needed on independently living, home-dwelling individuals in order to strengthen their functional capacity and thereby delay the need for more intensive and costly services, as well as the onset of 24-hour care.

Notably, the study relied on self-reported measures, which might increase the likelihood of social desirability bias. However, we used a validated question to identify individuals with mobility limitations [29] that is specifically suitable for population surveys, and corresponding information on adults’ functional capacity cannot be obtained from registers.

Our research utilized independent samples of the population collected at different time points. This approach lacked individual-level follow-up and prevented us from examining changes within individuals. Additionally, the projections are conditional, model-based estimates based on previously observed population-level trends and demographic assumptions rather than firm predictions. However, we used a multiple imputation method, commonly employed in population studies, to generate future projections [34–37]. This multiple imputation method is able to consider anticipated demographic changes in the age and sex structure of the population, which may improve the precision of the estimates. Relying on historical trends may raise concerns about the influence of short-term fluctuations, such as the increase observed in 2018–2020. However, the prevalence of mobility limitations remained high in 2022 and 2024, suggesting that this elevated level may not be merely a temporary anomaly. Moreover, population aging drives increases in mobility limitations, meaning projections are largely shaped by long-term demographic trends.

Finally, our study examined how mobility limitations change among older adults and how these changes relate to sex, age group, and educational attainment. However, a limitation of this study is that it does not identify the underlying mechanisms driving these changes. Further empirical and longitudinal research is needed to better understand the mechanisms across different sociodemographic groups.

## Conclusions

Our results highlighted a concerning upward time trend and projection in mobility limitations among the Finnish population aged 75 years and older. If effective preventive measures are not taken, the number of adults with mobility limitations is expected to almost double by 2040. These findings underscore the growing burden of mobility limitations in the aging population, which is likely to have significant consequences for their independence, healthcare utilization, and quality of life. The aging population, combined with an increasing prevalence of mobility limitations, will seriously strain healthcare systems and caregiving resources. Therefore, the independence of people with mobility limitations should be supported by increasing accessibility and other aspects of age-friendly society [61]. Also, we need to develop more targeted measures for older adults and preventive actions focused on improving opportunities for physical activity. Tailored strength training, balance exercises, and healthy nutrition are important. Supporting other aspects of functioning, such as social participation and cognition, may promote mobility and prevent mobility limitations [62, 63].

Our results highlighted that mobility limitations vary by educational level. Among highly educated individuals aged 55 years and older, the prevalence of limitations increased from 2018 to 2024 and is projected to continue rising to 2040. In contrast, those with a low or medium level of education only experienced a rise between 2018 and 2020, with prevalence stabilizing after 2020. These findings underline the importance of public health strategies to reduce socioeconomic disparities in mobility. Interventions should encourage workers in sedentary occupations to break up prolonged sitting and to integrate more physical activity into everyday routines, such as commuting by walking or cycling [56]. Additionally, increasing access to health coaching and preventive services for people with a low or medium level of education, particularly during crises, may help prevent their mobility limitations.

## Supplementary Information

Supplementary Material 1. Appendices A–C containing supplementary tables.

## Data Availability

The datasets analyzed in this study are not publicly available due to data protection regulations of the Finnish Institute for Health and Welfare (THL). Researchers may request access for scientific purposes through the Social and Health Data Permit Authority (Findata) by submitting a research proposal along with an approved user authorization application.
