# Supplementary material for: Increasing mobility limitations among older adults in Finland: time trends and projections during 2013–2040
Source: BMC Public Health. 2026 Aug 10;26:3224. doi: 10.1186/s12889-026-28941-6 (PMC13741546; doi:10.1186/s12889-026-28941-6)
Supplement: Supplementary file 1 [file 12889_2026_28941_MOESM1_ESM.docx]

**Additional file 1**

**Appendix A.** Trends from 2013 to 2024 and projections until 2040 for the prevalence of mobility limitations among adults aged 55 and older by age group

| **Total** | **55+**  %[95% CI] | **75+**  %[95% CI] | **55-64**  %[95% CI] | **65-74**  %[95% CI] | **75-84** %[95% CI] | **85+**  %[95% CI] |
| --- | --- | --- | --- | --- | --- | --- |
| 2013 | 11.7[11.4, 12.1] | 25.2[24.3, 26.1] | 5.4[5.0, 5.9] | 9.6[8.9, 10.2] | 18.4[17.5, 19.2] | 44.6[42.4, 46.8] |
| 2014 | 11.4[10.8, 12.0] | 25.0[23.7, 26.4] | 5.1[4.4, 5.9] | 9.1[8.1, 10.1] | 18.4[17.1, 19.7] | 44.6[41.2, 48.0] |
| 2015 | 11.3[10.8, 11.9] | 23.7[22.4, 25.0] | 5.6[4.9, 6.5] | 8.8[7.9, 9.8] | 16.7[15.5, 18.0] | 43.1[39.9, 46.4] |
| 2018 | 11.0[10.3, 11.7] | 22.9[21.7, 24.1] | 4.8[3.8, 6.0] | 9.0[7.9, 10.2] | 14.9[13.9, 15.9] | 42.0[39.1, 45.0] |
| 2020 | 13.5[13.0, 14.1] | 28.6[27.5, 29.7] | 6.2[5.4, 7.1] | 9.1[8.3, 10.1] | 20.7[19.7, 21.7] | 48.8[46.3, 51.3] |
| 2022 | 13.2[12.6, 13.7] | 26.8[25.8, 27.9] | 5.8[5.0, 6.8] | 8.7[7.8, 9.6] | 19.7[18.7, 20.7] | 47.2[44.5, 49.9] |
| 2024 | 13.9[13.3, 14.7] | 27.1[26.1, 28.1] | 6.1[5.2, 7.1] | 9.4[8.5, 10.5] | 19.7[18.7, 20.7] | 48.9[46.4, 51.3] |
| 2027 | 13.8[13.1, 14.5] | 27.9[26.3, 29.6] | 6.8[5.6, 8.3] | 9.6[8.0, 11.4] | 21.3[19.5, 23.2] | 50.2[47.4, 53.0] |
| 2030 | 14.8[14.1, 15.6] | 29.6[27.5, 31.7] | 6.7[5.5, 8.1] | 9.5[8.1, 11.1] | 22.8[21.1, 24.6] | 51.3[48.5, 54.1] |
| 2035 | 16.5[15.6, 17.4] | 31.7[29.6, 34.0] | 6.8[5.4, 8.5] | 9.1[7.7, 10.8] | 24.9[22.8, 27.1] | 53.3[49.7, 56.8] |
| 2040 | 17.7[16.7, 18.7] | 35.0[32.2, 37.8] | 6.8[5.3, 8.5] | 8.7[6.9, 10.9] | 26.9[24.2, 29.8] | 56.6[52.0, 61.0] |
| **Women** | **55+** %[95% CI] | **75+**  %[95% CI] | **55-64**  %[95% CI] | **65-74** %[95% CI] | **75-84** %[95% CI] | **85+**  %[95% CI] |
| 2013 | 13.3[12.7, 13.8] | 28.3[27.2, 29.5] | 5.4[4.8, 6.1] | 9.8[9.0, 10.8] | 19.5[18.4, 20.7] | 49.8[47.2, 52.4] |
| 2014 | 12.9[12.1, 13.7] | 27.9[26.1, 29.7] | 4.9[4.0, 6.0] | 9.4[8.1, 10.8] | 19.5[17.8, 21.3] | 48.8[44.7, 52.9] |
| 2015 | 12.9[12.1, 13.7] | 27.2[25.5, 29.1] | 5.5[4.6, 6.7] | 8.8[7.6, 10.2] | 18.7[17.0, 20.4] | 47.5[43.5, 51.5] |
| 2018 | 12.2[11.3, 13.1] | 26.1[24.4, 27.8] | 3.9[2.9, 5.3] | 8.8[7.4, 10.5] | 16.1[14.7, 17.5] | 46.1[42.4, 49.8] |
| 2020 | 14.9[14.2, 15.7] | 32.1[30.7, 33.6] | 6.0[4.9, 7.3] | 8.5[7.4, 9.7] | 22.5[21.2, 24.0] | 53.1[49.9, 56.3] |
| 2022 | 15.0[14.3, 15.8] | 30.2[28.7, 31.7] | 6.1[5.0, 7.4] | 9.1[7.9, 10.4] | 21.2[19.8, 22.6] | 51.6 [48.2, 54.9] |
| 2024 | 15.3[14.5, 16.1] | 30.5[29.2, 32.0] | 5.1[4.0, 6.3] | 9.1[7.8, 10.5] | 21.1[19.8, 22.5] | 53.8[50.7, 56.9] |
| 2027 | 15.6[14.5, 16.1] | 32.1[29.4, 34.9] | 5.1[3.9, 6.6] | 9.8[8.0, 11.9] | 22.9[20.9, 25.0] | 55.2[51.7, 58.7] |
| 2030 | 16.9[15.8, 18.0] | 34.1[31.0, 37.4] | 5.0[3.8, 6.5] | 9.5[8.1, 11.1] | 24.9[22.3, 27.7] | 56.0[52.1, 59.9] |
| 2035 | 18.8[17.5, 20.2] | 35.7[32.2, 39.5] | 5.1[3.7, 7.0] | 9.2[7.3, 11.5] | 27.0[24.2, 29.9] | 58.8[54.9, 62.6] |
| 2040 | 20.1[18.7, 21.6] | 38.2[33.7, 43.2] | 5.1[3.6, 7.2] | 8.9[6.9, 11.6] | 29.3[26.3, 32.4] | 62.4[57.0, 67.5] |
| **Men** | **55+**  %[95% CI] | **75+**  %[95% CI] | **55-64**  %[95% CI] | **65-74**  %[95% CI] | **75-84**  %[95% CI] | **85+** %[95% CI] |
| 2013 | 10.0[9.4, 10.5] | 20.4[19.1, 21.7] | 5.5[4.8, 6.2] | 9.3[8.4, 10.3] | 16.7[15.5, 18.0] | 33.8[30.2, 37.7] |
| 2014 | 9.6[8.8, 10.5] | 20.1[18.3, 22.1] | 5.4[4.3, 6.7] | 8.7[7.4, 10.3] | 16.6[14.8, 18.7] | 34.3[29.0, 40.0] |
| 2015 | 9.5[8.7, 10.3] | 18.0[16.3, 19.9] | 5.8[4.7, 7.0] | 8.9[7.6, 10.5] | 13.9[12.2, 15.7] | 33.3[28.3, 38.9] |
| 2018 | 9.6[8.6, 10.7] | 17.9[16.3, 19.6] | 5.6[4.1, 7.7] | 9.1[7.5, 11.0] | 13.3[11.9, 14.8] | 32.9[28.2, 38.0] |
| 2020 | 11.8[11.0, 12.6] | 23.3[21.8, 24.8] | 6.4[5.2, 7.9] | 9.9[8.6, 11.4] | 18.1[16.7, 19.6] | 40.0[35.9, 44.3] |
| 2022 | 11.0[10.2, 11.8] | 22.1[20.7, 23.6] | 5.6[4.5, 6.9] | 8.1[6.9, 9.6] | 17.8[16.4, 19.3] | 38.4[34.2, 42.8] |
| 2024 | 12.3[11.4, 13.2] | 22.2[20.8, 23.6] | 7.1[5.7, 8.9] | 9.8[8.4, 11.4] | 17.8[16.4, 19.2] | 39.0[35.2, 42.9] |
| 2027 | 12.3[11.2, 13.4] | 23.8[21.3, 26.5] | 6.9[5.4, 8.7] | 9.3[7.4, 11.4] | 19.3[17.1, 21.7] | 40.7[35.3, 46.4] |
| 2030 | 12.5[11.5, 13.6] | 25.8[23.1, 28.8] | 6.7[5.3, 8.5] | 9.3[7.2, 11.8] | 20.2[18.2, 22.3] | 42.9[37.5, 48.4] |
| 2035 | 13.9[12.6, 15.2] | 27.1[24.1, 30.5] | 6.8[5.1, 9.0] | 8.9[7.3, 10.8] | 22.4[19.9, 25.2] | 44.2[39.5, 49.0] |
| 2040 | 14.9[13.7, 16.1] | 29.2[25.0, 34.0] | 6.7[5.2, 8.5] | 8.9[7.1, 11.2] | 24.1[21.0, 27.4] | 47.7[43.0, 52.4] |

*Note.* CI = confidence interval.

**Appendix B.** Trends from 2013 to 2024 and projections until 2040 for the number of persons with mobility limitations and the projected population aged 55+ and 75+ in Finland

|  | **The number of persons with mobility limitations** | | **The projected population in Finland^a^** | |
| --- | --- | --- | --- | --- |
| **Total** | **55+** n  [95% CI] | **75+** n  [95% CI] | **55+** n | **75+** n |
| 2013 | 212579 [205786, 219565] | 117038 [113005, 121167] | 1811954 | 464396 |
| 2014 | 209658 [199109, 220690] | 119048 [112737, 125590] | 1837515 | 475901 |
| 2015 | 210997 [200447, 222027] | 113910 [107722, 120339] | 1862873 | 480675 |
| 2018 | 213045 [200294, 226501] | 117199 [111054, 123570] | 1935631 | 511969 |
| 2020 | 267566 [256640, 278881] | 156723 [150876, 162705] | 1980972 | 547835 |
| 2022 | 265307 [254479, 276522] | 161297 [154966, 167789] | 2013223 | 600850 |
| 2024 | 283393 [271775, 295425] | 177058 [170549, 183721] | 2041219 | 652831 |
| 2027 | 287441 [270334, 303159] | 199798 [188242, 211764] | 2064834 | 714942 |
| 2030 | 316604 [293357, 340633] | 226678 [210909, 243065] | 2090571 | 766645 |
| 2035 | 359991 [332978, 387986] | 263258 [245222, 281846] | 2153567 | 829237 |
| 2040 | 398643 [357022, 431547] | 306449 [282334, 331275] | 2229917 | 875981 |
| **Women** | **55+** n  [95% CI] | **75+** n  [95% CI] | **55+** n | **75+** n |
| 2013 | 131058 [125921, 136372] | 82674 [79315, 86119] | 988379 | 291661 |
| 2014 | 128977 [121063, 137327] | 82864 [77599, 88343] | 1000286 | 296983 |
| 2015 | 130306 [122322, 138730] | 81266 [76065, 86685] | 1012349 | 298392 |
| 2018 | 127777 [118740, 137399] | 81315 [76190, 86658] | 1046648 | 311981 |
| 2020 | 159487 [151488, 167831] | 105727 [100949, 110621] | 1068149 | 329289 |
| 2022 | 162875 [154568, 171546] | 107522 [102324, 112868] | 1083191 | 356268 |
| 2024 | 167650 [159292, 176363] | 117154 [111875, 122569] | 1097221 | 383551 |
| 2027 | 173037 [161636, 184858] | 133682 [122482, 145400] | 1107674 | 416446 |
| 2030 | 188734 [176414, 201503] | 151697 [137819, 166350] | 1118613 | 444968 |
| 2035 | 215558 [200804, 230891] | 171025 [154000, 189110] | 1145652 | 478942 |
| 2040 | 236911 [220054, 254465] | 191918 [169380, 216773] | 1175999 | 502156 |
| **Men** | **55+** n  [95% CI] | **75+** n  [95% CI] | **55+** n | **75+** n |
| 2013 | 81975 [77597, 86573] | 35253 [33046, 37568] | 823575 | 172735 |
| 2014 | 80239 [73454, 87579] | 36018 [32694, 39588] | 837229 | 178918 |
| 2015 | 80751 [74027, 88017] | 32868 [29654, 36346] | 850524 | 182283 |
| 2018 | 85397 [76629, 95051] | 35793 [32559, 39273] | 888983 | 199988 |
| 2020 | 107687 [100377, 115454] | 50847 [47597, 54248] | 912823 | 218546 |
| 2022 | 102202 [95226, 109621] | 54107 [50584, 57801] | 930032 | 244582 |
| 2024 | 115885 [107780, 124509] | 59803 [56126, 63649] | 943998 | 269280 |
| 2027 | 117468 [107410, 127970] | 70988 [63572, 78978] | 957160 | 298496 |
| 2030 | 121321 [111360, 131763] | 83128 [74373, 92578] | 971958 | 321677 |
| 2035 | 139837 [127347, 153028] | 95001 [84260, 106928] | 1007915 | 350295 |
| 2040 | 156761 [144037, 170114] | 109318 [93312, 127282] | 1053918 | 373825 |

*Note.* CI = confidence interval; ^a^Based on information from Statistics Finland (2024).

**Appendix C**. Trends for 2013–2024 and projections until 2040 for mobility limitations among adults aged 55+ by education

| **Total** | **Low** %[95% CI] | **Medium** %[95% CI] | **High** %[95% CI] |
| --- | --- | --- | --- |
| 2013 | 14.4[13.7, 15.0] | 9.2[8.6, 9.9] | 7.9[7.3, 8.5] |
| 2014 | 14.2[13.2, 15.3] | 9.3[8.3, 10.4] | 7.0[6.2, 7.9] |
| 2015 | 14.2[13.1, 15.2] | 9.8[8.8, 10.9] | 7.0[6.2, 7.9] |
| 2018 | 13.3[12.1, 14.6] | 9.7[8.7, 10.9] | 6.9[5.9, 8.1] |
| 2020 | 16.2[15.2, 17.2] | 12.8[11.9, 13.7] | 8.2[7.4, 9.1] |
| 2022 | 16.3[15.3, 17.3] | 11.3[10.4, 12.2] | 8.8[7.9, 9.6] |
| 2024 | 16.4[15.4, 17.4] | 12.1[11.0, 13.1] | 9.9[9.1, 10.8] |
| 2027 | 16.9[15.6, 18.3] | 12.9[11.6, 14.3] | 10.8[9.6, 12.1] |
| 2030 | 17.9[16.5, 19.4] | 13.8[12.0, 15.8] | 11.7[10.5, 13.0] |
| 2035 | 19.0[17.1, 21.1] | 14.9[13.0, 17.0] | 13.4[11.7, 15.2] |
| 2040 | 20.3[17.6, 23.2] | 15.9[13.4, 18.8] | 14.7[12.9, 16.7] |
| **Women** | **Low** %[95% CI] | **Medium** %[95% CI] | **High** %[95% CI] |
| 2013 | 15.9[15.0, 16.8] | 10.8[9.9, 11.7] | 9.7[8.8, 10.6] |
| 2014 | 15.2[13.8, 16.7] | 11.1[9.7, 12.6] | 8.7[7.5, 10.1] |
| 2015 | 16.5[15.1, 18.1] | 10.6[9.3, 12.0] | 7.9[6.8, 9.2] |
| 2018 | 14.9[13.3, 16.7] | 11.1[9.7, 12.6] | 8.0[6.7, 9.6] |
| 2020 | 17.0[15.7, 18.5] | 14.2[13.0, 15.5] | 10.0[8.8, 11.2] |
| 2022 | 19.4[18.0, 20.8] | 12.0[10.7, 13.3] | 10.4[9.2, 11.7] |
| 2024 | 17.5[16.2, 18.8] | 13.0[11.7, 14.4] | 11.8[10.5, 13.2] |
| 2027 | 18.3[16.7, 20.0] | 13.9[12.2, 15.7] | 12.6[11.1, 14.1] |
| 2030 | 19.4[17.7, 21.2] | 15.0[13.2, 17.0] | 14.0[12.5, 15.7] |
| 2035 | 20.8[18.7, 23.2] | 16.3[14.6, 18.3] | 16.0[14.0, 18.2] |
| 2040 | 21.9[19.9, 24.3] | 17.7[15.3, 20.4] | 17.1 [15.3, 19.2] |
| **Men** | **Low** %[95% CI] | **Medium** %[95% CI] | **High** %[95% CI] |
| 2013 | 12.7[11.8, 13.6] | 7.2[6.3, 8.2] | 6.0[5.2, 6.8] |
| 2014 | 13.1[11.6, 14.7] | 6.9[5.6, 8.5] | 5.1[4.1, 6.3] |
| 2015 | 11.5[10.2, 13.0] | 8.8[7.3, 10.5] | 6.0[4.9, 7.3] |
| 2018 | 11.7[10.1, 13.6] | 7.6[6.1, 9.5] | 5.6[4.1, 7.6] |
| 2020 | 15.2[13.8, 16.8] | 10.9[9.6, 12.5] | 6.4[5.4, 7.7] |
| 2022 | 13.1[11.8, 14.5] | 10.4[9.0, 11.9] | 6.9[5.9, 8.1] |
| 2024 | 15.1[13.6, 16.8] | 10.8[9.3, 12.6] | 7.9[6.7, 9.2] |
| 2027 | 15.5[14.1, 17.1] | 11.2[9.8, 12.8] | 8.4[7.2, 9.8] |
| 2030 | 16.0[14.1, 18.2] | 12.2[10.3, 14.4] | 9.0[7.7, 10.4] |
| 2035 | 17.2[15.0, 19.6] | 13.0[11.0, 15.2] | 10.4[8.9, 12.2] |
| 2040 | 18.7[16.0, 21.7] | 13.6[11.3, 16.4] | 12.0[10.2, 13.9] |

*Note.* CI = confidence interval.
